# Detecting clinically significant events through automated language analysis: Quo imus?

**DOI:** 10.1038/npjschz.2015.54

**Published:** 2016-01-06

**Authors:** Peter W Foltz, Mark Rosenstein, Brita Elvevåg

**Affiliations:** 1 Institute of Cognitive Science, University of Colorado, Boulder, CO, USA; 2 Pearson, Boulder, CO, USA; 3 Psychiatry Research Group, Department of Clinical Medicine, University of Tromsø, Norway; and Norwegian Centre for Integrated Care and Telemedicine, University Hospital of North Norway, Tromsø, Norway

We found the recent paper by Bedi *et al.*^1^ simultaneously exciting, heartening and, sadly, a bit discouraging. It shows that modern, statistical natural language processing (NLP) and machine-learning (ML) techniques can potentially be useful as a component of diagnosis, here predicting who among those at risk will eventually transition to full-blown psychosis. This result follows closely our own and others observations of the value of these techniques in, for example, discriminating patients with schizophrenia from controls,^2^ discriminating schizophrenia probands, first-degree relatives and unrelated healthy controls,^3^ differentiating those at high risk of psychosis from unrelated putatively healthy participants^4^ and in a candidate gene study linking language in general to underlying neurobiology,^5^ all quite encouraging outcomes.

Our disappointment is not directly with the Bedi *et al*.^1^ paper itself, but that we as a field are, after this long proving period, still at the ‘promising’ stage. This inertia arises from two primary factors. The first is owing to the use of small, often second-hand data sets produced for other studies, which severely constrains the NLP techniques that can be applied and the generality of the obtained results. The second is that the methodologies applied must become sufficiently assimilated into the field to be used effectively in analyses so as to provide valid, reliable measures of the constructs of interest. This understanding permits better linking of the appropriate features of language to the underlying etiologies of interest.

To realize the potential of the transformative next steps, we must routinely and systematically strive to obtain larger data sets containing multiple language samples from participants collected over time. This will allow quantifying the joint time course of the disease(s) and changes in language. Increased sample size further improves the methodologies, permitting moving beyond the less-reliable cross-validation to the use of the gold-standard for validating ML results, which is a ‘hold-out’ data set. In such an approach all modeling is conducted blind to the hold-out set, and when modeling is completed, the model is run on the held-out set to measure expected performance in the larger population, thereby ensuring generalization while lowering the risk of overfitting. At least as importantly, realistically sized data sets allow the application of larger combinations of more sophisticated NLP/ML techniques that move beyond the often used simple word-count features. This permits deeper characterization of more important aspects of language, such as semantic structures, discourse organization, as well as acoustic characteristics.^6^

From our perspective, Figure 3 from Bedi *et al*.^1^ is a beautiful, low-dimensional, small, incremental step toward our vision, which is that of a truly high-dimensional language-feature space with the potential to align with the aspirational goals of the NIMH Research Domain Criteria by employing language to locate and pinpoint those with severe mental illness at coordinates within this space. Once localized, the features that define the resulting hypothesized clusters can potentially be calibrated for use in early detection, continuously evaluating treatment and providing links to the biology underlying these diseases, simultaneously superseding our existing diagnostic categories. But this vision is only achievable with purpose-designed studies containing sufficiently large populations with a mix of both healthy participants and individuals sampled across multiple categories of diagnostic groups. Our field must become versed in the use of more powerful applications of NLP/ML techniques and offer more reproducible methodologies. These results, taken with others, are sufficiently encouraging so that it is now time for us to move beyond ‘promising’.
